# Burning of Brides in South Asia

**DOI:** 10.12669/pjms.37.2.4053

**Published:** 2021

**Affiliations:** Tehreem Sultan, School of Law, Cardiff University, Cardiff, United Kingdom. Email: SultanT@cardiff.ac.uk

Dear Editor,

I would like to draw your attention to the heinous act of burning of brides in the South Asian region. The burning of brides is a challenging medico legal and human rights problem of low-and middle-income countries.^1^ Domestic violence has long lasting effects on human health, with multiple behavioral and psychological disorders. Women are the majority of its victims.^2^ The five forms of domestic violence include physical violence, sexual violence, emotional abuse, honor killing, and dowry-death or the bride burning practice which is carried out in South Asia, including India, Nepal, Afghanistan, Pakistan and Bangladesh.^3^ This heinous act of burning brides has been identified historically with a particular part of the world, South Asia, and is persistent mainly in India.^1^

Bride-burning occurs when young females are murdered by either their husband or the family for her family’s refusal to pay additional dowry, which is a major violation of fundamental human rights. There are multiple factors which place the bride at risk of burning including; lack of education, poverty, unemployment, dowry tradition, customary conditions, early marriage, and inadequate legislative framework. Even in the 21^st^ century, females face discrimination and inequality at home and the workplace and are moreover at times even denied the right to be born, since many families selectively abort baby daughters. Aborting female fetuses is practically and socially considered acceptable in some parts of the Indian Subcontinent. This practice of female feticide is primarily due to paying dowry to the future bridegroom of a daughter^4^.

The dowry culture is a leading cause of bride burning mainly in low-income developing countries^1^. The dowry system dates back to Greco-Roman times and refers to the transfer of goods such as gold, jewelry, land property, cash money, motor vehicles, televisions and property to a bridegroom from a bride’s family as a condition of the marriage^5^. In some cases, the dowry goods payment system continues throughout a married woman’s life to conciliate her husband. If the bride’s parents are unable to pay the money or goods to the bridegroom, the dowry system becomes the main cause of suicide or burning of the bride or young wife. In 2015, 7634 women died due to dowry harassment, representing approximately 21 cases per day in India specifically^6^. Despite the legislation, dowry system is still common in many regions of South Asia.

The majority of dowry deaths occur within the first few years of marriage. The common types of dowry deaths involve drowning, poisoning and hanging, or using flammable liquid such as gasoline and setting alight, leading to death by fire^6^. While this violates the right to life, freedom from torture and degrading treatment, and is discrimination between the genders, this practice is a crime and has been treated as culpable homicide. Furthermore, if it is proven, it can be punished by up to lifelong imprisonment or the capital punishment depending on countries which have not abolished the Death Penalty as per the Universal Declaration of Human Rights.

There is a strong need to establish strict legislation and increase awareness, while educating the communities, to amend the cultural and social norms in which there remains a request for dowry. These factors can reduce the practice of bride price and the violent act of bride burning. Moreover, human right organizations in collaboration with the international community must implement strict policies, to eradicate the dowry culture and the frequent cases of burning brides, and minimize violence against females in various parts of the world.
